# A fugacity model assessment of ibuprofen, diclofenac, carbamazepine, and their transformation product concentrations in an aquatic environment

**DOI:** 10.1007/s11356-018-3485-x

**Published:** 2018-11-05

**Authors:** Tuomas M. A. Nurmi, Toni K. Kiljunen, Juha S. Knuutinen

**Affiliations:** Department of Chemistry, University of Jyvaskyla, P.O. Box 35, FI-40014, Jyväskylä, Finland

**Keywords:** Pharmaceuticals, Transformation products, Multimedia model, Environmental fate, Stratified lake, Phototransformation, Wastewater treatment plant

## Abstract

An updated version of FATEMOD, a multimedia fugacity model for environmental fate of organic chemicals, was set up to assess environmental behaviour of three pharmaceuticals in northern Lake Päijänne, Finland. Concentrations of ibuprofen, diclofenac, and carbamazepine were estimated at various depths at two sites: near a wastewater treatment plant and 3.5 km downstream the plant. When compared with environmental sampling data from corresponding depths and sites, the predicted concentrations, ranging from nanograms to hundreds of nanograms per litre, were found to be in good agreement. Weather data were utilised with the model to rationalise the effects of various environmental parameters on the sampling results, and, e.g. the roles of various properties of lake dynamics and photodegradation were identified. The new model also enables simultaneous assessment of transformation products. Environmentally formed transformation product concentrations were estimated to be at highest an order of magnitude lower than those of the parent compounds, and unlikely to reach a detectable level. However, a possibility that conjugates of ibuprofen are present at higher levels than the parent compound was identified. Simulation results suggest that environmental degradation half-lives of the inspected contaminants under stratified lake conditions are in the range of some weeks to months.

## Introduction

A growing variety of chemicals are produced by modern industry and used all over the world (O’Neill 1998). Expanded search efforts and advanced analytical technologies have uncovered the environmental presence of a plethora of anthropogenic chemicals, and many of them are known to be, or are considered potentially harmful. A global effort to assess their risks and to reduce the amounts of contaminants ending up in the environment has been ongoing for decades. New threats are occasionally uncovered, such as traces of pharmaceutical substances (Heberer 2002; Lindholm-Lehto et al. 2015) and other emerging pollutants (von der Ohe et al. 2011). For some chemicals, transformation product (TP) and metabolite concentrations and toxicities (la Farré et al. 2008; Donner et al. 2013) may even exceed those of the parent compounds. On the other hand, many conjugated metabolites can deconjugate back to their respective parent compounds in the environment (Celiz et al. 2009; Azuma et al. 2017).

One of the major entry routes of emerging contaminants to the environment seems to be in municipal sewage waters through wastewater treatment plants (WWTPs), which often have limited trace chemical removal efficiency (Evgenidou et al. 2015). The continuous output of emerging pollutants from WWTPs may cause them to be constantly present in the environment at notable concentrations. Pharmaceutical removal and transformation in water treatment, and occurrence of TPs in the environment, has been reviewed by Evgenidou et al. (2015), who note the lack of systematic toxicological data on TPs. The methods and challenges of analysing conjugates and TPs have been further discussed by Brown and Wong (2015) and Fatta-Kassinos et al. (2011). Great variation by chemical is also seen in the behaviour at WWTPs. While some compounds transform into harmless or potentially harmful products, others might pass through WWTPs unchanged, or bind in the WWTP sludge, with the latter also laying concerns on later utilisation of the sludge.

As environmental monitoring resources are constrained, infrequent sampling of the pollutant levels may result in misrepresentation of the concentration levels (Gevaert et al. 2009). One approach to investigating longer trends and average levels of contaminants is measuring concentrations from sediments or passive samplers (Martínez Bueno et al. 2016). Another possibility to build a better understanding of the situation is modelling the contaminants’ environmental fate. Fugacity-based environmental fate modelling of organic chemicals, as published by Mackay (2001), is a well-known approach to modelling of environmental contaminants. Similar methods have been applied to e.g. mountain environment (Westgate and Wania 2013), drainage basin (Wania et al. 2000), and global (MacLeod et al. 2011) scale assessment of environmental fate.

In the present work, a new version of FATEMOD (Paasivirta et al. 2009), an environmental fate model based on Mackay’s models, was set up to inspect pharmaceutical levels in northern Lake Päijänne, central Finland, reported by Lindholm-Lehto et al. (2015). Three pharmaceuticals with notable environmental levels, anti-inflammatories ibuprofen (IBU) and diclofenac (DCF), and antiepileptic carbamazepine (CBZ) were selected for modelling, primarily targeting the two sampling sites with most data available, the so-called sites 1 and 4. Since the measurements were carried out during a period of several months, acquiring weather data for the corresponding period allowed assessing the concentrations’ dependencies on environmental parameters. Fugacity models typically sacrifice some complexity and accuracy in exchange for modest data requirements, making it possible to inspect a variety of scenarios with relatively little effort. Traditionally fugacity models have been used to inspect persistent compounds, but the relatively short persistences of pharmaceuticals require a somewhat different model composition. Therefore, the new model version was developed to allow for environments that consist of any number of compartments, chemicals, and arbitrarily definable intra-system flows. The model was augmented to support transformation reactions between chemicals, as well. By taking advantage of these features, the requirements for modelling pharmaceuticals in a stratified lake environment were identified and a corresponding model environment set up. The main objective of the research was to verify the applicability of the model by comparison to available experimental data. The model was then to be used to assess the effects of various environmental parameters on pharmaceutical concentrations and to rationalise the strong variations and patterns observed in the experimental data. Secondary aim was to estimate the significance of typical TPs of the pharmaceuticals in the modelled environment.

## Methods

### The environmental fate model

The fugacity approach (Mackay 2001) is used to find out the chemical equilibrium between environmental compartments. A fugacity capacity of a chemical is calculated for each compartment, and an equilibrium fugacity of the chemical is obtained for the whole environment. A Level I model consists of a fixed amount of chemical partitioned into the compartments. Level II treats the environment as steady-state and at internal equilibrium, with chemicals forming and degrading, and being transported into and out of the system by advection. On Level III intra-system flows between the compartments are included and Level IV adds temporal variance, allowing retrieving the concentrations as a function of time.

The chemical concentration in a compartment is solved by multiplying fugacity *f* with the compartment’s fugacity capacity *Z*. All the concentration dependent flows, including advection, intra-system transport, and reactions, can be expressed as *D* values which, when multiplied with fugacity, give the molar flow rate. FATEMOD uses fugacity, fugacity capacity, and reaction rate calculation methods corresponding to the Level III model, but adds the temperature dependence of chemical properties, and also supports acids and bases.

The new version, FATEMOD-Q, was developed using Qt^Ⓡ^ application framework (https://www.qt.io) and Eigen linear algebra library (https://eigen.tuxfamily.org). Matrix-based calculation of the fugacity equilibrium is used: A matrix of *D* values is constructed, with each column corresponding to a target compartment and each row to a source compartment, and with diagonal values containing the flow out from the compartment. Solving the equation 𝔻**f** = **b**, where 𝔻 is the matrix and **b** are the constant flows into each of the compartments, gives equilibrium fugacities for the compartments. To enable calculation of transformation reactions, inter-reacting chemicals are grouped into reaction chains, and the matrix for each chain has dimensions *nm* × *nm*, where *n* and *m* stand for number of compartments and number of different chemicals, respectively. Each chemical in each compartment is represented by a column and a row, and the matrix is therefore freely expandable, allowing any number of reacting chemicals in any number of compartments. Adding new environmental media types, e.g. urban films (Csiszar et al. 2012) and indoors (Palm Cousins 2012), to the model is also straightforward, requiring at minimum only a method for calculating fugacity capacity and a density for the new media type. The new version uses a modified method for acid and base dissociation, based on Trapp and Matthies (1998).

### Selection of transformation products, reaction rates, and chemical properties

Biodegradation and photolysis are the important transformation reactions for IBU, DCF and CBZ (Koumaki et al. 2017; Gröning et al. 2007; Radke and Maier 2014). All the compounds can undergo photolysis, at least in the presence of photosensitisers in natural waters (Jacobs et al. 2011; Fatta-Kassinos et al. 2011), and IBU and DCF biodegrade. However, CBZ apparently biodegrades only in soils (Radke and Maier 2014; Li et al. 2013). As the number of possible TPs is high for each IBU (Ferrando-Climent et al. 2012; Vione et al. 2011), DCF (Jewell et al. 2016; Agüera et al. 2005), and CBZ (Kosjek et al. 2009), a subset of them was selected for inspection. The parent compounds, the selected TPs, and their relevant properties are listed in Tables 1, 2, and 3.

**Table 1:** Structures and physicochemical properties of ibuprofen and selected transformation products

id	Structure	Molecular weight	Water solubility	log *K*_OW_	*K*_OC_/*K*_OW_	p*K*_a_
		(g/mol)	(g/l, pH 7)	(pH 7)		
IBU		206.29	1.13^a^	1.23^a^	0.46^b^	4.91^c^
IBAP		176.26	5.37^d^	1.34^d^	*(0.46)*	*(4.91)*
IBU-CBX		236.26	130.13^d^	0.14^e^	*(0.46)*	3.97^e^
IBU-2OH		222.28	137.36^d^	−0.29^e^	*(0.46)*	4.63^e^

^a^Fini et al. (1986). ^b^Scheytt et al. (2005). ^c^Antonić and Heath (2007). ^d^Estimated using http://vcclab.org/web/alogps, Tetko et al. (2005). ^e^Ferrando-Climent et al. (2012)

**Table 2:** Structures and physicochemical properties of diclofenac and selected transformation products

id	Structure	Molecular weight	Water solubility	log *K*_OW_	*K*_OC_/*K*_OW_	p*K*_a_
		(g/mol)	(g/l, pH 7)	(pH 7)		
DCF		296.15	1.82^a^	1.14^a^	1.0^b^	4.15^c^
CPAB		231.68	3.28^d^	0.77^e^	*(1.0)*	*(4.15)*
5HDQI		310.13	38.17^d^	0.60^d^	*(1.0)*	3.60^f^

^a^Fini et al. (1986). ^b^Scheytt et al. (2005). ^c^Antonić and Heath (2007). ^d^Estimated using http://vcclab.org/web/alogps, Tetko et al. (2005). ^e^Schulze et al. (2010). ^f^Estimated using PerkinElmer ChemDraw^Ⓡ^ Professional 15

**Table 3:** Structures and physicochemical properties of carbamazepine and selected transformation products

id	Structure	Molecular weight	Water solubility	log *K*_OW_	*K*_OC_/*K*_OW_	p*K*_a_
		(g/mol)	(g/l, pH 7)	(pH 7)		
CBZ		236.27	0.075^a^	2.7^b^	5.4^c^	13.9^d^
AI		179.22	0.04^e^	3.50^e^	*(5.4)*	5.45^f^
AO		195.22	0.10^e^	3.21^e^	*(5.4)*	*(13.9)*

^a^Kadam et al. (2009). ^b^Mersmann et al. (2002). ^c^Scheytt et al. (2005). ^d^Lindholm-Lehto et al. (2015). ^e^Estimated using http://vcclab.org/web/alogps, Tetko et al. (2005). ^f^Perrin (1965)

Degradation and transformation data are widely available in the literature for all three parent compounds. However, detection and quantification limit data, and quantitative information on the formed TPs are scarce due to the lack of model compounds (Agüera et al. 2005). TP formation is also strongly dependent on conditions (Evgenidou et al. 2015; Collado et al. 2012; Ferrando-Climent et al. 2012). The selected pseudo-first order reaction half-lives for each compound are shown in Table 4. The values in the table are equal to the total half-life when combined, for example $\left [(200 \mathrm {\ h})^{-1}+(50 \mathrm {\ h})^{-1}\right ]^{-1} = 40 $ h for IBU. Yield of each TP is the ratio of its formation half-life to the total half-life of the parent compound. For the TPs with no half-lives listed, total reaction rates of the parent compounds have been applied.

**Table 4:** Selected photo- and biotransformation reaction half-lives

Phototransformation			Biotransformation		
Reaction		Half-life (h)	Reaction		Half-life (h)
IBU	→ IBAP	200^a^	IBU	→ IBU-CBX	70^c^
	→ other	50^a,b^		→ IBU-2OH	35^c,d^
IBAP	→ other	21^b^		→ other	7^c,d^
			IBU-CBX	→ other	8^d^
			IBU-2OH	→ other	7^d^
DCF	→ CPAB	44^e,f^	DCF	→ 5HDQI	92^c,g^
	→ other	4.4^e^		→ other	11^c^
			5HDQI	→ other	13^g^
CBZ	→ AI	10 000^h,i^			
	→ AO	100 000^h,i^			
	→ other	1 000^h^	(No CBZ biotransformation)		
AI	→ other	450^i^			
AO	→ other	450^i^			

^a^Jacobs et al. (2011). ^b^Ruggeri et al. (2013). ^c^Kunkel and Radke (2008). ^d^Collado et al. (2012). ^e^Bartels and von Tümpling (2007). ^f^Estimation based on Agüera et al. (2005). ^g^Gröning et al. (2007). ^h^Andreozzi et al. (2002). ^i^Estimation based on Donner et al. (2013)

IBU can transform photochemically via various reaction paths (Vione et al. 2011; Jacobs et al. 2011). 4-isobutylacetophenone (IBAP), a photo-TP with multiple formation routes and known adverse effects (Ruggeri et al. 2013), was selected as an IBU photoproduct (Table 1). Although carboxyl ibuprofen (IBU-CBX) and 2-hydroxyl-ibuprofen (IBU-2OH) are not the most toxic IBU bio-TPs (Marco-Urrea et al. 2009), they are present with the highest concentrations and have a potential environmental impact (Zwiener et al. 2002; Collado et al. 2012).

Two important DCF photoproducts are 8-chloro-9H-carbazole-1yl-acetic acid (Fatta-Kassinos et al. 2011; Agüera et al. 2005) and 2-[(2-chlorophenyl)amino] benzaldehyde (CPAB) (Schulze et al. 2010), of which the latter was selected for modelling (Table 2). The bio-TP *p*-benzoquinone imine of 5-hydroxydiclofenac (5HDQI) (Gröning et al. 2007) was also selected.

Biodegradation is of little effect for CBZ (Stamatelatou et al. 2003), and CBZ conjugates were also ignored, even though their role is significant at least in WWTP processes (Lindholm-Lehto et al. 2015). Thus included were two photo-TPs with evident toxicity, acridine (AI) and acridone (AO) (Donner et al. 2013) (Table 3).

Among the various environmental parameters affecting degradation reactions are aerobic-anaerobic conditions, humic compounds and other organic material in water, nitrate concentration, and pH (Lahti and Oikari 2011; Fatta-Kassinos et al. 2011; Andreozzi et al. 2002; Koumaki et al. 2015). To avoid unmanageable complexity, these were ignored in the reaction rate setup. However, the reaction rates were temperature corrected as described by Sinkkonen and Paasivirta (2000). While Koumaki et al. (2015) and Koumaki et al. (2017) have estimated photolysis to be an important pharmaceutical degradation path, their measurements were done in accordance to OECD guideline 316 (2008), which represents photolysis in the surface layer of water only. However, Bartels and von Tümpling (2007) have reported the diminishing effect of depth on photolysis. The sediment type dependence of biodegradation has also been investigated, but according to Radke and Maier (2014), such variations are dominated in importance by exchange dynamics between water and sediments.

Chemical properties required by the present application of FATEMOD-Q model are molecular weight, water solubility, octanol-water partitioning coefficients (*K*_OW_), and p*K*_a_, of which water solubility and *K*_OW_ can optionally be temperature dependent. Due to the minimal vapour pressure of the inspected compounds, the gas-phase concentrations are known to be very low, and no air compartment is necessary. Soils were also left out as their only input in current scenario would have been from air. However, soil compartments could be added if one wanted to, for example, inspect the effects of sludge utilisation (Topp et al. 2008). As pointed out by Mackay et al. (2014) among others, a fixed (organic carbon-water)-(octanol-water) partitioning proportions (*K*_OC_/*K*_OW_) ratio traditionally used in fugacity models is often not sufficient. Therefore, the current model can optionally use a chemical-specific value instead of the default 0.41. Temperature-dependent water solubility data were available for all three parent compounds as well as temperature dependent *K*_OW_ for IBU and DCF. Where no parameters for TPs were available, computational values were used (methods listed in table footnotes). For IBAP, CPAB, and AO no p*K*_a_ could be acquired, so the parent compound’s value was used instead. The *K*_OC_/*K*_OW_ values of parent compounds were also used for each of the TPs. Parameters used are listed in Tables 1, 2, and 3, with all temperature dependent values at 25 ^∘^C. FATEMOD-Q groups neutral and dissociated forms of a molecule together. Therefore, the solubilities and *K*_OW_ s are shown with value representing both forms at pH 7, in contrast to the more conventional *K*_OW_ of neutral species and water solubility at pH ≪ p*K*_a_. The water solubility is corrected following Trapp and Matthies (1998) and *K*_OW_ by calculating it as the fraction of octanol solubility and water solubility at pH 7. The protolysation calculations in the model were validated by building a Level I model environment simulating IBU partitioning pH dependency measurements by Hiller and Šebesta (2017) and comparing the results. Although low pH partitioning differed greatly, the results at pHs ≥ 5.8 agreed well, suggesting that the calculations are adequate for environmental applications.

### Model setup

**Fig. 1:**
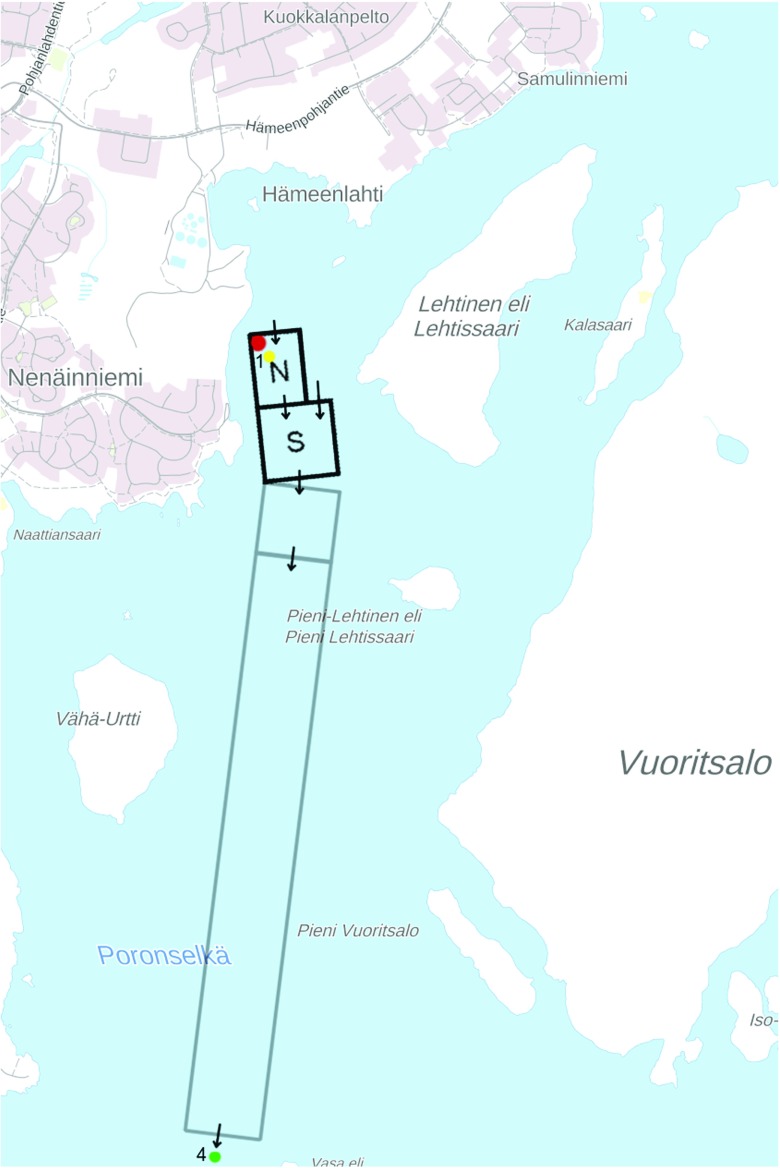
The effluent release point (at the depth of approx. 3.5 m), sampling site 1, and sampling site 4 are marked by red, yellow and green dots, respectively, on the map. Northern (N) and southern (S) lake model areas are located upstream the 3.3 km long transport model area. The short and long arrows depict water velocities of 1 cm/s and 2 cm/s, respectively. (map: National Land Survey of Finland 2017, “Taustakartta 1:20 000”, 11/2017, Creative Commons BY 4.0)

The model environment was constructed to represent a part of northern Lake Päijänne in central Finland, shown in Fig. 1. The specific focus was set on the Lindholm-Lehto et al. (2015) sampling sites 1 and 4, since most data were available from these measurements downstream a WWTP: concentrations at the depths of 1 and 5 m (site 1), and 1, 10, and 20 m (site 4). The stratified lake environment was set up based on meteorological data from Finnish Meteorological Institute, water temperatures from Kuha et al. (2016) and the Finnish environmental information system Hertta, and other lake data from Palomäki et al. (2014). Rough comparisons of environmental concentrations with temperature, total water flow, and wind data during the samplings showed that wind speed had clearly the strongest effect on the levels, with the rest possibly being completely overshadowed by the effluent variations. The measurements of Lindholm-Lehto et al. (2015) were therefore categorised into two groups based on the average wind speed in the area during three days preceding the samplings. Three samplings, denoted “weak wind,” were found to have an average wind < 2 m/s. Four samplings, denoted “stronger wind,” had an average wind of 3–5 m/s. None of the weak wind samplings took place during, or shortly after, the high disturbance mixing events identified by Kuha et al. (2016). The weak wind samplings were the primary subjects of investigation, as they were expected to be less complicated to model, and the model environment was built to simulate relatively calm conditions. The stronger wind samplings were analysed with an effort to identify possible adjustments needed to improve the model performance.

**Fig. 2:**
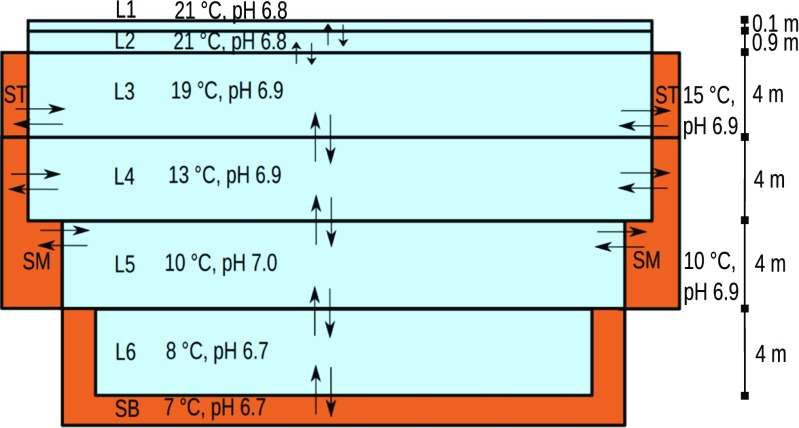
A cross-section of the northern (N) and southern (S) model area (not to scale). Northern layers (NLs) are 200–150 m and southern layers (SLs) 300–200 m wide. L6 and SB are only present in the southern area

The area near the emission point was split into northern (N) and southern (S) part, each with a length of 300 m, as shown in Fig. 1. The water in each area was divided depthwise based approximately on the temperature layers present, denoted L1–L6, as shown in the cross-section of the area in Fig. 2. The rationale for L1 and L2 division was to gain the ability to set customised photolysis rates near the lake surface. 2 cm layers of bottom sediments at three different depths (Sediment Bottom SB, Sediment Middle SM, and Sediment Top ST) were also included in the model. The complete vertical mixing durations of adjoining water layers were set following Schwarzenbach et al. (2003): Lake vertical turbulent diffusivity parameters are listed to be 0.1–10^4^ cm^2^*s*^− 1^ in mixed layer and 10^− 3^–10^− 1^ cm^2^*s*^− 1^ in deep water. 1 cm^2^*s*^− 1^ was selected for layers over thermocline and 0.1 cm^2^*s*^− 1^ under. To get an estimate of the vertical mixing velocity, diffusivity parameters were divided by the vertical distance of the midpoints of two adjacent layers. The vertical mixing velocity was further multiplied with the interface area between layers to get volumetric flow rate. When the layer volumes were divided by the rates, the resulting durations for complete mixing of adjacent layers were L1 to L2 0.014 h, L2 to L3 22 h, L3 to L4 1 600 h, and 16 000 h for L4 to L5 and L5 to L6.

For sediment-water transport flows and sediment organic carbon content (6%), parameters by Paasivirta et al. (2009) were used. The role of dissolved organic material (DOM) has been reported (Kukkonen and Oikari 1991), but was left unexplored since bioavailability was not the target of this work. However, the DOM values of 9 mg/l reported by Huovinen et al. (2003) were applied to suspended sediments: they were set to have a 25% organic carbon content and a volume of 0.036‰ of the water volume in each compartment. Biotransformation was set to take place in the bottom sediments. The effect of depth on photolysis (Bartels and von Tümpling 2007) was accounted for by using lower reaction rates in deeper water layers. The difference of secchi depths (Lake Goitsche: 8 m, Bartels and von Tümpling (2007), Päijänne: 2 m, Lindholm-Lehto et al. (2015)) suggests that also UV penetration differs significantly. Based on Huovinen et al. (2003), full reaction rate was used for 0–0.1 m, 10% of the rate for 0.1–1 m, 1% for 1–5 m, and no photodegradation for lower layers. Data reported by Lindholm-Lehto et al. (2015) were used to set up an effluent stream to compartment NL3, with a flow of 1700 m^3^/h and IBU, DCF, and CBZ concentrations of 1 μg/l, 1.5 μg/l, and 0.05 μg/l, respectively. Temperature layer data (Kuha et al. 2016) suggest that although most of the time mixing between water layers is expectedly weak, under some conditions the water is completely mixed to depths of at least 10 m. Preliminary dynamic modelling showed that for the most important N-S model compartments, when starting from zero levels, equilibrium pharmaceutical concentrations are reached in some days. Since the environmental data could be used to verify the stability of the environment during the samplings, assessing the environment with a steady-state equilibrium (“Level III“) model and relatively limited vertical mixing over thermocline was therefore reasonable.

Sampling site 4 lies approximately 3.5 km downstream of sampling site 1, as shown in Fig. 1, and a separate model environment was constructed to inspect the behaviour of the compounds during transport between the sites. The transport environment consisted of a single set of L1–L4 water compartments identical to SLs, and a bottom sediment compartment with mass transfer between L3 and L4. Two versions were set up: one for both weak and stronger wind scenarios. Water flow velocity was set to 1 cm/s for the weak and to 3 cm/s for the stronger wind scenario, with other parameters identical to the N-S model. The transport model was applied in two phases: first, the contaminant levels from S water layers were passed through a 300 metre model environment, and afterwards the 300 metre results through a 3 km long but otherwise identical environment. In addition, the 3 km transport model environment was further applied recursively to find out degradation timescales and peak concentrations for various TPs. The recursion was done by repeatedly using the previous output concentrations as new input levels until the TP levels plateaued. Possible role of IBU conjugates capable of transforming back to IBU (Celiz et al. 2009) was examined by including a pseudo-chemical, IBU conjugate, in the transport model, with 3 times the initial IBU concentration and a deconjugation reaction to IBU with a 2 h half-life.

A trend of effluents sinking during the transport was observed in the measured levels (Lindholm-Lehto et al. 2015). This was taken into account in the transport model input concentrations by averaging over the concentrations in the corresponding S layer and in the layer above it. In addition, preliminary results suggested that weaker horizontal and stronger vertical mixing would describe the stronger wind scenario better. This was accounted for by omitting the dilution in S area and using NL concentrations as a starting point instead, and selecting tenfold vertical mixing rates.

## Results and discussion

### Comparison of modelled and measured data

**Fig. 3:**
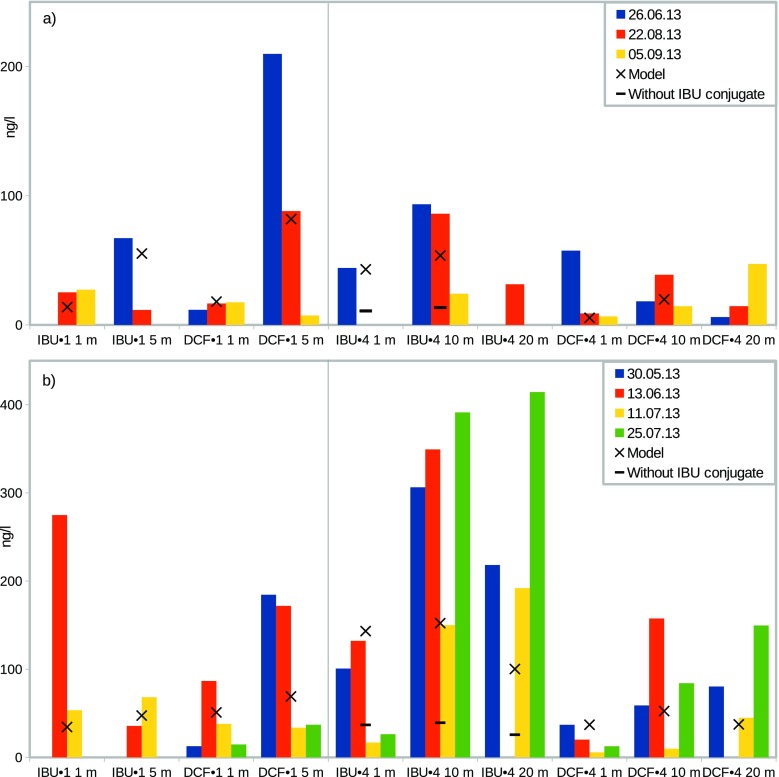
IBU and DCF concentrations at sampling sites 1 and 4 measured by Lindholm-Lehto et al. (2015) and corresponding model results, where **a** average wind < 2 m/s, and **b** average wind 3–5 m/s

First, we describe how the model results compare with the environmental sampling data. Since the results show that a predominant fraction of IBU and DCF are partitioned into the water compartments, the modelled IBU and DCF concentrations in water are presented with the measured environmental levels in Fig. 3. We compare the N model values to site 1 levels and transport model values to site 4 levels. The bar diagram in Fig. 3a (upper panel) shows the chemical concentrations on the three sampling dates with the weak-wind conditions, and the crosses represent the modelled values at the best-corresponding layer depths. For the site 1 sampling depths of 1 m and 5 m, we select the NL2 and NL3 values for comparison, whereas for site 4, the one- and ten-metre sampling depths are compared with the L2 and L4 of the transport model, respectively. The 20 m sampling depth is beyond the modelled water layers in this case. With the present set of modelling parameters, the output values can be considered reasonably representative. On site 1, the concentrations remain higher for the effluent input layer NL3 than for the surface layer NL2 where photodegradation prevails. On site 4, the L4 IBU concentrations are higher than at the surface, also in agreement with the measurements. The IBU levels in effluents are subject to major instability (Lindholm-Lehto et al. 2015), which is reflected in stronger temporal variation of the measured IBU levels than the DCF levels. Peculiarly, IBU levels also tend to be higher after transport, while DCF levels are generally lower at site 4 than at site 1. With the inclusion of conjugates of IBU in the model, the increase of IBU concentration from site 1 to site 4 proves similar to the experimental data. Thus, a portion of IBU might escape detection at site 1 but emerge after deconjugation at site 4. A varying fraction of conjugates could also explain some of the variation at site 1. This is in contrast to Azuma et al. (2017), who did not find IBU levels to be affected by enzymatic deconjugation at corresponding levels. However, IBU and IBU conjugate behaviour in water treatment is complicated and condition dependent (Lindholm-Lehto et al. 2015; de Graaff et al. 2011; Zwiener et al. 2002). Thus the idea of deconjugation, or some other phenomenon with similar effects, in different environmental conditions and after a different WWTP, seems still acceptable.

The evident temporal variation in the concentrations becomes amplified under the stronger wind conditions, as seen in Fig. 3b (lower panel). With weak wind speeds, the dilution taking place in S area corresponds to the dilution during transport from site 1 to site 4 in the environment. However, stronger wind sampling results suggest relatively weaker horizontal dilution. This is likely caused by higher water velocities and shorter residence times in the area between the sampling sites. Thus, stronger wind model results are in the same range with the corresponding sampling results when dilution in S area is omitted. The inclusion of IBU conjugates is again an important factor to better match the experimental levels by increasing the site 4 values. The result of modified water flows can be seen by comparing the modelled site 1 values for IBU and DCF. For example, the surface DCF values increase from 18 to 51 ng/l and the effluent release layer values drop from 82 to 69 ng/l between the weak and stronger wind scenarios. The effect of modified input flows and dilution can be seen in the site 4 surface concentrations, where the IBU value increases from 43 to 143 ng/l, for example. For the deeper sampling depths, the best model correspondence was found by comparing the 10 and 20 m levels with transport model L3 (1–5 m) and L4 (5–9 m) values, respectively. This mapping adjustment was made assuming yet an extra degree of sinking for the chemicals in the stronger wind conditions.

**Fig. 4:**
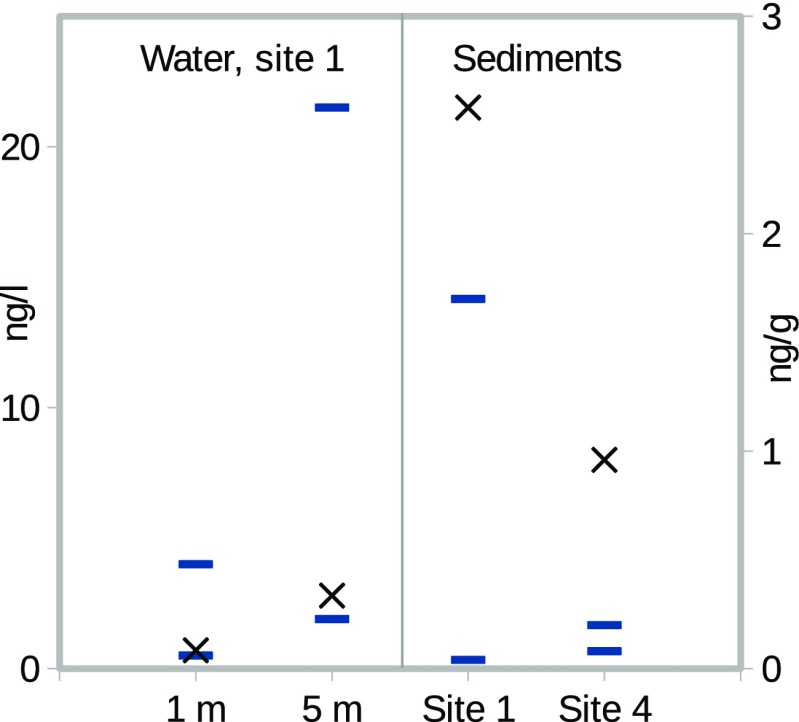
Modelled CBZ concentrations (crosses) compared with the measured minimum and maximum levels (line segments) in water (left) and sediments (right). Measured data from Lindholm-Lehto et al. (2015)

According to the model results, most of CBZ is bound in sediments. The highest concentrations in water have been measured at sampling site 1 (Lindholm-Lehto et al. 2015). Thus, we show a comparison of modelled and measured CBZ concentrations in water (sampling site 1) and in sediments (sites 1 and 4) in Fig. 4. The experimental variation is represented by the line segments, and the modelled values (crosses) are seen to settle in between the minimum and maximum levels in the site 1 water volume. In sediments, the concentrations are predicted slightly above the experimental range, which suggests that binding to sediments is somewhat overestimated. A possible reason could be the *K*_OC_/*K*_OW_ parameter, and an adjustment to its value to a lower binding would also increase the CBZ content in water to better settle towards the measured average.

A subset of the modelled N-S area results is shown in Table 5. We see that when using default model parameters, the predicted maximum concentrations for IBU, DCF and CBZ (55.2, 81.9, and 2.76 ng/l, respectively) are in agreement with the measured concentration ranges at site 1 (Fig. 3). A rough assessment of the degree of mixing and dilution during the first hundreds of metres can be made based on the differences between the water layers. This gives an estimation, that the typical area with pharmaceutical levels in tens of nanograms per litre can range to a few square kilometres. Expectedly, the modelled sub-thermocline concentrations (data not shown) remained low, as transport through thermocline is limited in a calm environment. The modelled L4, L5, and L6 concentrations were generally 2, 4, and 4 orders of magnitude lower than L3 concentrations, respectively. For IBU and DCF the modelled ST concentrations are 4 to 5 orders of magnitude smaller than the ones measured by Lindholm-Lehto et al. (2015). Possible reasons include a too high biodegradation rate, the failure of the pH corrections in an actual environmental scenario, and differences between the sediments in current area and the ones used in *K*_OC_ measurements by Scheytt et al. (2005). Another kind of explanation could also be deduced from the fact that modelled concentrations in L3 suspended sediments (data not shown) are within one order of magnitude of the measured sediment levels for all three parent compounds. It is possible that the sediment samples collected using fortnightly emptied sedimentation tubes are not representative of the modelled well mixed bottom sediments at equilibrium, exhibiting contaminant levels between the modelled bottom and suspended sediments instead. As the sedimentation to SM and SB takes place from L4–L6, their concentrations were correspondingly 2–3 and 5 orders of magnitude lower than ST concentrations, respectively. This suggests that pharmaceuticals are consistently to be found in sediments near the effluent point, and less often, probably when the weather or other external factors have induced strong-enough mixing, in sediments elsewhere. This is in line with sampling site 3 and 5 sediment results of Lindholm-Lehto et al. (2015). Modelled concentrations in the sediments after 3.5 km transport for both IBU and DCF, which were not detected at site 4, were approximately 1 × 10^− 3^ ng/g.

**Table 5:** Modelled pharmaceutical concentrations in selected N and S model compartments

(ng/l)	NL2	NL3	SL2	SL3	ST (ng/g)
IBU	13.8	55.2	10.7	26.5	1.25 × 10^− 4^
IBAP	3.82 × 10^− 2^	1.12 × 10^− 2^	3.92 × 10^− 2^	1.06 × 10^− 2^	2.00 × 10^− 8^
IBU-CBX	2.98 × 10^− 9^	1.19 × 10^− 8^	7.18 × 10^− 9^	2.99 × 10^− 8^	8.23 × 10^− 6^
IBU-2OH	5.05 × 10^− 9^	2.02 × 10^− 8^	1.22 × 10^− 8^	5.07 × 10^− 8^	1.38 × 10^− 5^
DCF	18.0	81.9	13.4	38.9	4.07 × 10^− 4^
CPAB	1.78 × 10^− 1^	6.39 × 10^− 2^	1.73 × 10^− 1^	5.82 × 10^− 2^	1.87 × 10^− 7^
5HDQI	8.14 × 10^− 9^	3.86 × 10^− 8^	1.97 × 10^− 8^	9.69 × 10^− 8^	3.01 × 10^− 5^
CBZ	7.00 × 10^− 1^	2.76	5.48 × 10^− 1^	1.33	2.58
AI	3.54 × 10^− 5^	1.01 × 10^− 5^	3.67 × 10^− 5^	9.61 × 10^− 6^	8.17 × 10^− 5^
AO	3.86 × 10^− 6^	1.10 × 10^− 6^	4.00 × 10^− 6^	1.05 × 10^− 6^	4.98 × 10^− 6^

### Sensitivity and parameter dependency of the results

To study the sensitivity of the model results, the model parameters and estimations were varied and the effects on N-S levels were surveyed. The variations and their effects are listed in Table 6. Among the factors with most effect is the mixing and dilution of the WWTP effluents, determined especially by the water flow through the area. In addition to water flow variations, the high parent compound levels in NL3 and SL3 were notably affected by photolysis changes. IBU and DCF levels in ST were affected especially by biodegradation rate and log*K*_OW_ related changes, and CBZ levels in ST by the amount of suspended sediments. Various environmental parameters affect especially the lower concentrations relatively much, although the absolute concentration differences are usually only a small fraction of the parent compounds’ peak concentrations. Prime examples are IBU and DCF in sediments: in base results, less than a hundredth of a percent of their total mass is partitioned in sediments, and even when the sediment results are strongly affected by some parameters, the absolute differences are so small that they are unlikely to cause a notable effect on the peak concentrations in water.

**Table 6:** Selected effects of various changes to the N-S model environment

Parameter	Change	Notable effects
Water layer mixing	L1 $\leftrightarrow $ L2 $\leftrightarrow $ L3 $\leftrightarrow $ L4 rates to 10%	parent compounds, bio-TPs: L3, ST +, elsewhere $^{-}_{-}$
	Doubled between all layers	parent compounds, bio-TPs: L3, ST −, all compounds: L1, L2 $^{+}_{+}$, elsewhere $^{\hspace {0.08cm}+}_{++}$
Water flow velocity	Western side 0.5 cm/s	max IBU 106 ng/l, DCF 155 ng/l, CBZ 5.3 ng/l, ST $^{+}_{+}$, SL1, SL2, SL3 parent levels $^{+}_{+}$, other levels elsewhere $^{\hspace {0.08cm}+}_{++}$
Water sinks deeper	Water flowing NL1 → SL2, NL2 → SL3 etc.	SL4 IBU 33 ng/l, DCF 50 ng/l, CBZ 1.7 ng/l, SL1, SL2, SL3 $^{\hspace {0.08cm}-}_{--}$, ST average $^{-}_{-}$, other southern and sediment compartments $^{+}_{+}{}^{+}_{+}$.
Effluent input	Effluent input flow to NL2	parent, photo-TPs: L1, L2 $^{+}_{+}{}^{+}_{+}$, parents elsewhere $^{\hspace {0.08cm}-}_{--}$, photo-TPs elsewhere $^{\hspace {0.08cm}+}_{++}$, bio-TPs everywhere $^{\hspace {0.08cm}-}_{--}$, total mass in area − 40%
Chemical parameters	IBU and DCF log*K*_OW_+ 2	bio-TPs $^{+}_{+}{}^{+}_{+}$, IBU, DCF: L6 $^{-}_{-}$, sediments $^{+}_{+}{}^{+}_{+}$
Temperatures	All compartments 20 ^∘^C	photo-TPs: L1, L2 −, other water layers +, photo-TPs, CBZ: sediments +, IBU, DCF: sediments $^{-}_{-}$
	4 ^∘^C higher over thermocline, 4 ^∘^C lower under	photo-TPs $^{+}_{+}$, IBU, DCF: SM, SB $^{+}_{+}$
Photolysis depth effect	25% rate in L2, 10% rate in L3	photo-TPs $^{\hspace {0.08cm}+}_{++}$, parent compounds −, bio-TPs −
Degradation reactions	No photolysis	L1, L2 IBU, L3 DCF +, L1, L2 DCF $^{+}_{+}$, no photo-TPs
	No biodegradation	IBU, DCF: sediments $^{+}_{+}{}^{+}_{+}$, no bio-TPs
TPs in WWTP effluent	IBU bio-TPs 5% of IBU input	max IBU bio-TPs 2.8 ng/l, sediments $^{+}_{+}$, 10^3^ − 10^9^ times higher in water
Parent→TP conversion	TP-forming reaction rates doubled	photo-TPs $^{\hspace {0.08cm}+}_{++}$, bio-TPs $^{+}_{+}$
Suspended sediments	Doubled	bio-TPs $^{+}_{+}$, all ST, SM levels $^{+}_{+}$, SB $^{\hspace {0.08cm}+}_{++}$, CBZ, AI, AO: L5, L6 $^{+}_{+}$
	Removed	bio-TPs $^{-}_{-}$, CBZ, AI, AO: L5, L6 $^{-}_{-}$, sediments $^{\hspace {0.08cm}-}_{--}$, others: sediments $^{-}_{-}$

+: + 1–10%, $^{+}_{+}$: + 10–100%, $^{\hspace {0.08cm}+}_{++}$: + 100–1000%, $^{+}_{+}{}^{+}_{+}$: > + 1000%, −: −1–10%, $^{-}_{-}$: −10–50%, $^{\hspace {0.08cm}-}_{--}$: −50–100%

The response to minor amplification of the photolysis shows the importance of the depth dependency. Directly using photodegradation half-lives measured following OECD guideline 316 (2008) for the complete water body would produce totally different model results, demonstrating the necessity of setting specific reaction rates for each compartment. Lindholm-Lehto et al. (2015) have claimed that the lower concentrations closer to the surface are caused by photolysis. However, the model results suggest that major reasons include the effluent being output to deeper layers of the lake (Krogerus et al. 2013) and the relatively limited transport between the layers. The modelled molar flow of IBU in the water mixing flow between NL3 and NL2 is almost 50 times higher than the sum of IBU photolysis in NL1, NL2, and NL3. Since the used half-lives assume continuous sunshine, photolysis is also at its maximum level in the results. As it is unlikely to have a significant effect on pharmaceutical molecules at the depths of 10 and 20 m (Bartels and von Tümpling 2007; Huovinen et al. 2003), the concentration profile in deep water is probably a direct result of lake water dynamics. Specifically, this might be caused by either effluents flowing deeper due to bottom topography or seiching, or suspended sediments intensifying the descent of the pharmaceuticals. On the other hand, photolysis should definitely not be ignored in modelling, as its role is evident especially near the surface. Without photolysis, differences up to 23% can be observed in modelled DCF concentrations at 0.1 m depth with the relatively small N-S model environment. In addition, the magnitude of photodegradation increases further proportionally to the environment size with longer residence times in the model area.

Lindholm-Lehto et al. (2015) have reported the volumetric WWTP effluent flow to vary in the range of 1300–1900 m^3^/h and effluent IBU, DCF, and CBZ concentrations in the ranges 0–4 μg/l, 0.5–2.8 μg/l, and 0.01–0.09 μg/l, respectively. The environmental concentrations predicted by the model are directly proportional to these parameters. Intermediate values were used in the model, indicating that by varying the emissions in the measured range, the model results would span from one third of the current results to double values. Furthermore, the modelling was done with the assumption that water flowing into area is free from contaminants. As this might not always be the case (Lindholm-Lehto et al. 2015, 2016), background levels could also have a notable effect on environmental concentrations downstream. The sensitivities listed in Table 6 can also be used to estimate the effects of the applicable environmental parameters on sampling results. The available data do not permit any rigorous statistical analysis, but e.g. the smaller concentration differences at depths of 1 and 5 m at site 1 (Fig. 3a vs b) can be partially attributed to increased vertical mixing. Wind and water dynamics are also a probable cause of the variations observed in sediments. The sensitivities highlight the importance of accounting for environmental conditions when planning and assessing samplings. Various environmental parameters possibly affecting pharmaceutical degradation were ignored, but based on the reported effects on reaction rates, the magnitude of their effect on the results would be, at highest, similar to the one of increased photolysis.

### The peak levels and toxicity of transformation products

As shown in Table 5, the highest photo-TP water concentrations in the the modelled N-S area can be found near the surface, and conversely bio-TP concentrations in water peak closer to the sediments. Although the modelled sediment levels for IBU and DCF, and therefore also their bio-TPs, are possibly too low, the ratio of parent compounds and TPs is likely correct. When applying the 3 km transport model environment recursively, with 1 cm/s water velocity and no dilution, the total pharmaceutical amount is approximately halved after a month in the transport environment. The peak concentrations for most TPs were found to be after approximately 20 km, corresponding to 23 days. For most TPs, the levels were in pg/l range at highest. However, IBAP and CPAB showed maximum concentrations in ng/l range, approximately 1/10 and 1/50 of the NL parent compound levels (IBAP including the effect of the assumed IBU conjugate).

The toxicity values for AI and AO documented by Donner et al. (2013) (at lowest 0.07 mg/l) are not expected to be reached in the modelled environment. Schulze et al. (2010) have shown CPAB to be more toxic than DCF, starting to affect at approximately an order of magnitude lower concentrations, although even the lowest No Observed Effect Concentration for DCF, 1 μg/l, is three orders of magnitude higher than the peak CPAB concentrations in the model results. IBAP has been found in sewage waters at a maximum concentration of 40 ng/l, but not in a river with a limit of detection of 7 ng/l (Zorita et al. 2007). The model results suggest that unless considerable amounts of IBAP is formed in the WWTP processes, the levels in the examined environment are unlikely to reach the detection or acute toxicity levels, which are at lowest in the μg/l scale (Natali Sora and Fumagalli 2017). Same applies for IBU bio-TPs formed in the environment.

In general, the environmentally formed TP concentrations, as well as the concentrations of their parent compounds, are expected to remain clearly lower than any direct toxicity threshold measured for them. However, information on them is valuable when assessing mixture toxicities and cumulative chronic stresses on ecosystems (Vasquez et al. 2014). The transport modelling suggests also that if dilution would remain limited in an otherwise similar aquatic environment, IBAP and CPAB peak levels could reach a detectable level, but only dozens of kilometres downstream. They could create there a stress comparable to the one from the initial parent compound levels. However, a TP emission from a WWTP with a hundredth concentration of the parent compound would raise environmental levels of various TPs to ng/l range, indicating the importance of effective water treatment when limiting the risk of pharmaceutical TPs. Nevertheless, in this case the environmental formation would cause TP levels to decrease noticeably slower, extending the affected area.

## Conclusions

To our knowledge, the first fugacity model assessment of pharmaceuticals and transformation products in the environment was presented. The variations in measured levels demonstrate the feasibility of such modelling: reproducing each of the sampling results using more accurate and specific models would require vast amounts of data, many of which are not obtainable. With the current model, the scenario could be assessed with a modest amount of parameters required. Even unsteadier conditions could be accounted for with justifiable customisations of the model environment. Using intermediate emission levels, almost every modelled water result was between the highest and the lowest value at the corresponding site and depth, and often close to their average. This suggests that the model sufficiently describes the major processes affecting pharmaceutical behaviour in the inspected environment, and the parameters used were valid approximations.

Sensitivity analysis was used to identify the effect of various parameters on the model result. The total modelled pharmaceutical amount in the area was most strongly affected by changes related to water flow. The model results suggest that environmental sampling results are notably affected by variations in water dynamics and photolysis, and that they are the primary causes for the concentration profiles observed in the measured data. The difference in agreement of predicted and measured concentrations between IBU and DCF pointed out the presence of a previously unidentified phenomena affecting IBU behaviour. IBU conjugates capable of transforming back to IBU are a plausible explanation. Such conjugates are known to similarly affect behaviour of various other pharmaceuticals; therefore it is clearly necessary to account for metabolites when estimating the stress caused by measured environmental concentrations of pharmaceuticals. An estimate of various TPs in the modelled environment indicated that the TPs themselves are not a major concern, since their environmentally formed concentrations only barely reach detectable levels.

Fugacity models are suitable for planning samplings, screening, and similar purposes. Ideally, the final aim could be the ability to use such model as a partial replacement for environmental samplings. In the present work, our model was proficiently applied as a platform for combining environmental sampling results with other data to gain new information. Treating photodegradation as a function of depth was found to be essential when assessing environmental fate in a stratified humic lake. However, IBU and DCF levels in sediments differed orders of magnitude from measured ones, pointing out the need to better understand their binding and interaction with sediments. Assessment with a Level IV model would also allow inspecting the temporal development of concentrations more closely. In addition, the effects of dynamically changing environmental parameters and the documented seasonal variation of pharmaceutical levels are interesting subjects for the modelling in future.
